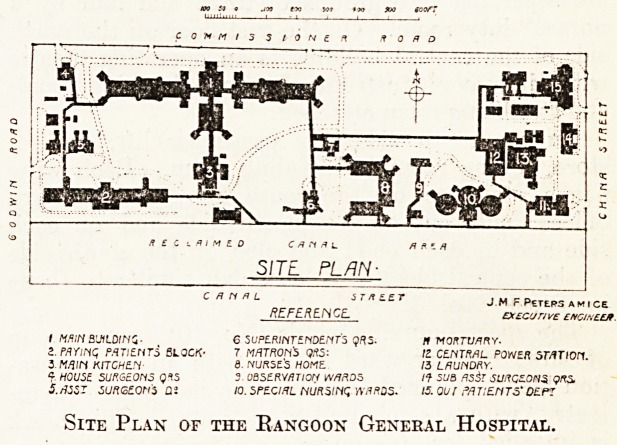# General Hospital, Rangoon

**Published:** 1915-02-13

**Authors:** 


### HOSPITAL ARCHITECTURE AND CONSTRUCTION.

### General Hospital, Rangoon.

This, the latest example of a complete general hospital in the East, was designed for the Local Government of Rangoon by Mr. J. M. F. Peters, A.M.I.C.E., the executive engineer.

The site, approximately 1,700 feet from east to west and 600 feet from north to south, contains an area of about 23½ acres. If, as is intended, the reclaimed canal area is added to the hospital grounds, the total area will be about 32 acres, and the site will be bounded on all four sides by roads.

The hospital comprises twenty-six separate buildings, the most important of which are shown on the block plan by figures from 1 to 15.

The main building, No. 1, contains four storeys, the lowest being a half-basement, which is used as an ambulatory, and contains also $x$-ray and electro-therapeutic rooms, with stores and offices.

The building is divided up into four separate blocks, a centre block with a ward block on each side and a third ward block projecting from the south side. The central block contains offices for the staff and admission rooms for patients on the ground floor. On the first floor is the operating-room suite, comprising the theatre, about 30 ft. by 20 ft., with a north window about 23 ft. wide; and instrument and sink rooms communicating with the theatre. On the east side of the corridor is the anæsthetic room and medical officers' changing room, and on the west side the sterilising room, apparatus room, and students' changing room. On the east side of the central staircase is the $x$-ray room and dark room, and on the west side the electro-therapeutic room. On the south side of the main corridor connecting this block with the ward blocks are two small surgical wards.

The ward blocks are divided in the centre by a block of separation wards, day rooms, ward kitchen, scullery, sisters' room, and linen room. On one side of this centre block is a ward for twenty-four beds; on the other a ward for thirty beds. All round each ward block is a verandah 9 ft. 7 in. wide, provided with windows that can be closed in case of need.

The sanitary offices are in projecting towers with cross-ventilated lobbies, and contain, besides the necessary sink rooms and w.c.s, shower baths as well as ordinary bathrooms. These last are very small, and the baths are not, as is always the case in this country, accessible from both sides. It would be impossible to carry a helpless patient into these bathrooms. Corresponding with the sanitary towers, the buildings projecting to the south are nurses' rooms.

The octagonal wing at the north-west angle of the central block in each case contains a winding staircase, the block at the south-west is a pantry, and the other two contain w.c.s and lavatories.

The ward block at the south contains on each floor two separation wards for sixteen beds each, divided by a wall and having windows on one side only.

The rest of the block is occupied by nurses' duty

Site Plan of the Rangoon General Hospital.

The First Floor, showing the Paying Patients' Block.

room, pantry, scullery, day room, and by sanitary offices in projecting towers at the south-west and south-east angles. A verandah similar to that round the front wards runs all round this block.

### Block for Paying Patients.

The paying-patients' block (No. 2) is three storeys high, the lowest storey being a half-sunk ambulatory. In this block the staircase and administrative offices are in the centre, and the wards, seven single rooms on each side, are in the wings. The wards are separated into groups of three and four by a nurses' duty room. On the first floor on the north side of the central building is an operating theatre with sink room, instrument room, changing room, and sterilising room attached.

On the south side is a passenger lift, general store, dining room and reading room. Each wing has a staircase on the south side, and sanitary offices built out on the north side and on each side and at each end; and also on the south side of the central block is a verandah similar to those in Block No. 1.

The special nursing wards (No. 10) form a group of three circular ward blocks with an administration block, planned evidently for the sake of symmetry, with a semicircular enclosure. This block is intended for friendless and destitute persons, mostly natives, who are brought to the hospital in a moribund state.

Two of the wards are 50 ft. 10 in. in diameter, the third being 35 ft. 10 in.; all are surrounded by an open verandah. The administration block contains rooms for changing clothes for patients of both sexes, clinical laboratory, drug-store, linen room, pantry, ward kitchen, and nurses' room. The sanitary offices are in projecting wings, with a passage open at the sides connecting them to the verandahs.

The observation wards (No. 9) contain five single-bedded wards for cases suspected of being infectious.

The Nurses' Home (No. 8) is partly two and partly three storeys in height, and provides accommodation for the assistant matron, housekeeper, six sisters, and forty-four nurses. The sisters have separate rooms, the nurses are provided with cubicles. Separate dining rooms and sitting rooms are provided for the nurses and sisters, the kitchen offices being on the top floor.

The kitchen block is placed to the south of the south wing of Block 1. Besides the main kitchen, which is fitted with apparatus of the European type, there is a "chula" kitchen for caste people. There is also a refrigerating plant and cold-storage chamber. Block 13 is the laundry block, which is equipped with modern disinfecting, washing, drying, and ironing apparatus, with separate machinery for dealing with soiled linen. The laundry is capable of dealing with 3,000 pieces daily. Adjoining is a refuse destructor.

### Laundry and Power Station.

No. 12, the central power-station, contains the boiler house and engine room, the electrical substation of the Rangoon Electric Tramway and Supply Company, a motor garage and electrical store, and a linen store. The latter is placed here in order that advantage may be taken of the steam and hot-water pipes for keeping the linen dry in the wet season.

Separate quarters are provided for the medical superintendent (No. 6), the matron (No. 7), the house surgeons (No. 4), assistant house surgeons (No. 5), and sub-assistant surgeons (No. 14).

The out-patient department (No. 15) is a one-storey building at the north-east angle of the site. It comprises the usual consulting and examining rooms for male and female patients, two small operation rooms for minor operations, and a dispensary.

The completed hospital affords accommodation normally for 443 patients, which, in cases of necessity, can be increased to 480, and has cost about £560 per bed.

## Figures and Tables

**Figure f1:**
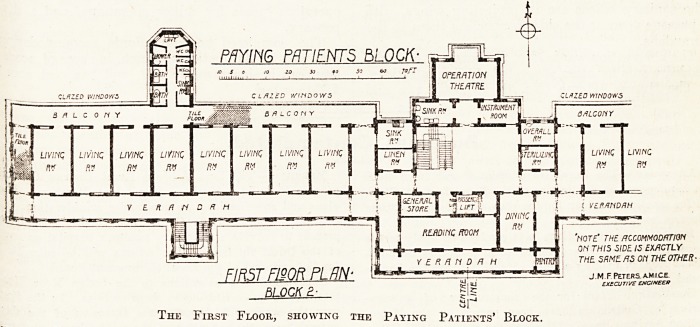


**Figure f2:**